# Fabrication of Homogeneous High-Density Antibody Microarrays for Cytokine Detection

**DOI:** 10.3390/microarrays3040282

**Published:** 2014-12-09

**Authors:** Ingeborg Hospach, Yvonne Joseph, Michaela Kathrin Mai, Nadejda Krasteva, Gabriele Nelles

**Affiliations:** 1 Materials Science Laboratory, Sony Deutschland GmbH, Hedelfinger Strasse 61, 70327 Stuttgart, Germany; E-Mails: michaela.mai@eu.sony.com (M.K.M.); nadejda.krasteva@eu.sony.com (N.K.); gabriele.nelles@eu.sony.com (G.N.); 2 Institute of Electronic and Sensor Materials, Technische Universität Bergakademie Freiberg, Gustav-Zeuner-Strasse 3, 09599 Freiberg, Germany; E-Mail: yvonne.joseph@esm.tu-freiberg.de

**Keywords:** microarray, protein, antibody, cytokines, spotting, hydrogel, biosensor, fluorescence readout

## Abstract

Cytokine proteins are known as biomarker molecules, characteristic of a disease or specific body condition. Monitoring of the cytokine pattern in body fluids can contribute to the diagnosis of diseases. Here we report on the development of an array comprised of different anti-cytokine antibodies on an activated solid support coupled with a fluorescence readout mechanism. Optimization of the array preparation was done in regard of spot homogeneity and spot size. The proinflammatory cytokines Tumor Necrosis Factor alpha (TNFα) and Interleukin 6 (IL-6) were chosen as the first targets of interest. First, the solid support for covalent antibody immobilization and an adequate fluorescent label were selected. Three differently functionalized glass substrates for spotting were compared: amine and epoxy, both having a two-dimensional structure, and the NHS functionalized hydrogel (NHS-3D). The NHS-hydrogel functionalization of the substrate was best suited to antibody immobilization. Then, the optimization of plotting parameters and geometry as well as buffer media were investigated, considering the ambient analyte theory of Roger Ekins. As a first step towards real sample studies, a proof of principle of cytokine detection has been established.

## 1. Introduction

Breath analysis is an interesting and motivating field of research; most recently, it has been found that dogs can smell lung cancer with high probability if trained accordingly [1]. Furthermore, lung diseases like asthma and chronic obstructive pulmonary disease (COPD) show similar somatic symptoms: the airways are inflamed but different medication is required. To distinguish between the diseases, classical diagnostic methods like imaging, spirometry, analysis of bronchoalveolar lavage (BAL), and invasive bronchoscopy are currently applied [2,3,4]. The vision for tomorrow, however, will be as in many medical fields the point-of-need diagnosis, a non-invasive breath analysis in order to spare patients invasive examinations, to increase the specificity of analysis, and to reduce costs. Critical high concentrations of nitrogen monoxide (NO) in breath, a potential marker for asthma, are already detected by chemical gas sensors, but the gaseous fraction of the breath varies strongly depending on personal habits [5,6]. On the other hand, and similar to other body fluids, like blood, urine, and saliva, monitoring of the cytokine pattern in exhaled breath condensate (EBC) can contribute to the identification and diagnosis of diseases [7,8,9,10,11]. Depending on the kind of disease, the body reacts with a certain distinct immune response, and thus different cytokines are present in specific amounts and different cytokine patterns are characteristic, as described for COPD and asthma in Barnes *et al.* [8].

The challenge of EBC analysis will be that the characteristic cytokines are only present in very low concentrations (<pg/mL) [10]. Thus, the detection mechanism of the antibody microarray has to be very sensitive in case a pre-concentration of the sample needs to be avoided. This required high sensitivity of antibody microarrays is a general problem in non-invasive diagnostics, and a solution would be of advantage for many different applications. Several groups are working on this challenge applying different strategies to improve the limit of detection (LoD) of antibody microarrays. For example, Cretich *et al.* detected cytokines in the pg/mL range by fluorescence enhancement using a mirror-like coating of the substrate [12]; while Pla-Roca *et al.* realized a special separation of detection antibodies to reduce cross-reactions and hence to detect different biomarkers, including cytokines in the ng-pg/mL range [13]. 

While DNA microarrays are already used in several fields of research and diagnostics, protein microarrays are not yet well established in diagnostics. One reason is that in contrast to DNA, it is not trivial to maintain the antibody’s structure and function when it is immobilized on a solid surface [14]. However, different functionalized surfaces have been found to be suitable for protein immobilization. Especially 3D polymer/hydrogel modified surfaces or porous nitrocellulose surfaces are often used as substrates in the field of protein microarrays [14].

There are fast test kit immunoassays on the market, *i.e.*, antibody-based assays, which are very well established for diagnostics, giving a fast yes/no answer due to a well-established and validated biomarker. Low analyte concentrations are rarely detected with these immunoassays; however, this is not a must for e.g., pregnancy tests, borreliosis tests, and many others. For example, the hormone human chorionic gonadotropin (hCG) in urine, indicating a pregnancy, is detected in the ng/mL range. In a biochemical laboratory, however, protein microarrays for diagnostic purposes are still not well accepted in the medical field, and the Enzyme Linked Immunosorbent Assay (ELISA) is still the golden standard also for the measurement of cytokine concentrations. Nevertheless, only a few types of antibody and cytokine microarrays are available on the market for research purposes and only few are, for example, used in the field of biomarker discovery and validation [15]. Table 1 summarizes several properties and features of selected commercial arrays.

**Table 1:** Overview of selected commercial ready-to-use cytokine antibody microarrays for laboratory use only (n.a. = not available).

Company	Product/Application	Solid Surface	Readout Mechanism	Spot Size/Distance	Sensitivity (LoD)
Full Moon BioSystems	Cytokine Antibody Array	3D polymer on glass substrate	Cytokine biotinylation, fluorescence readout	260–280 µm/600 µm	30–100 µg of total protein
KeraFAST^®^	FAST Quant^®^ Human cytokine assay	3D nitro-cellulose polymer on glass substrate	Sandwich format, fluorescence readout	150 µm/300 µm	Down to pg/mL range
RayBiotech	Antibody Array (different series)	Glass slide or membrane	Sandwich format or cytokine biotinylation, fluorescent or chemiluminescence readout	120 µm (glass), 1 mm (membrane)/n.a.	Down to pg/mL range

When looking at the antibody microarrays for cytokine detection that are on the market (Table 1), it can be noticed that they all tend to have a similar set-up and geometry. All suppliers offer a 3D matrix on a solid support either for covalent immobilization or physisorption of antibodies, possibly due to the option of a denser loading and a longer stability for the antibodies. The readout strategies are based on fluorescence or chemiluminescence without a further signal amplification step. The spot sizes of the arrays are usually between 100–300 µm, with a spot to spot distance of several hundred microns (~278 spots/cm^2^, in case of 600 µm spot-to spot distance). This most probably results from the given spotting instrumentation, reader/scanner, and experiences with commonly used DNA arrays. The sensitivity of said antibody microarrays is given in the pg/mL to ng/mL range, depending on the target and according to the suppliers. 

The aim of this work is the development of a high-density fluorescent immunoassay with the option to further amplify the signal. To make cytokine detection in potential low concentrations, such as later in exhaled breath condensate, possible, we chose to fabricate a planar 2D or 3D support, non-bead based, antibody microarray. As interesting targets and starting point for experiments we selected Tumor Necrosis Factor alpha (TNFα, M_W_ = 17 kDa) and Interleucin-6 (IL-6, M_W_ = 20 kDa) as they are, among other cytokines, involved in asthma and COPD development [8]. To improve the sensitivity of the antibody microarray, small spot sizes were targeted as already postulated by Roger Ekins [16,17]. Ekins’ ambient analyte theory concludes that the reduction of spot sizes improves signal-to-noise ratios as signal density and not the total sum of signal is relevant to define the lower LoD. To print smallest possible spots of antibodies on a solid support, automated nanoplotting can be used. However, to fabricate small spot antibody microarrays in a reliable and reproducible way, several parameters e.g., spotting buffer and surface chemistry for the immobilization of capture antibody as well as the wetting behavior have to be optimized.

**Figure 1:**
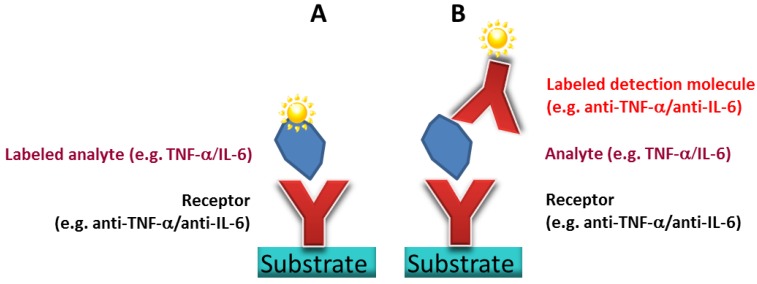
(**A**) Scheme of direct antigen capture immunoassay on a glass substrate. For detection, the analyte is labeled directly. (**B**) Scheme of sandwich-type immunoassay on a glass substrate. The labeled second antibody is used for analyte detection.

Finally, Figure 1 illustrates two possible schemes of the targeted antibody microarray structure: detection of the antigen by a direct analyte labeling (A), or alternatively a sandwich-type antibody microarray detecting the analyte with a labeled second antibody (B). The first results will be shown to demonstrate the proof of the principle and to draw a first conclusion. 

## 2. Experimental Section

### 2.1. Spotting Solutions

Millipore water was used for the preparation of buffers and solutions. TNFα and IL-6 antibodies with different fluorescent labels—Fluorescein Isothiocyanate (FITC), Allophycocyanin (APC), and Phycoerythrin (PE)—were used for spotting optimization and spotting control (Anti-Human TNF alpha, clone MAb11, labeled with -FITC, -APC, or -PE and Anti-human IL-6, clone MQ2-13A5, labeled with -FITC, -APC, or -PE, all from eBioscience, Frankfurt am Main, Germany). 

Unlabeled capture antibodies of rabbit anti-hTNFα and rabbit anti-hIL-6 antibodies were used for the detection of cytokines (all within the ELISA Development Kits, PeproTech, Hamburg, Germany).

The spotting buffer composition for the dilution of the antibodies was optimized and compared with a commercial spotting buffer (see further details in the Supplementary Materials). As spotting buffer a 10 mM sodium phosphate buffer (Merck KGaA, Darmstadt, Germany), pH 7 with 1% glycerol (Sigma-Aldrich, Munich, Germany) resulted in the best spot morphology and reproducibility for selected solid supports and antibody combinations and was used for all experiments if not otherwise stated. The antibody concentration and its influence on the spotting results were investigated and the results are also given in the Supplementary Materials. All antibodies were diluted with the optimized spotting buffer to a final concentration of 25 µg/mL if not otherwise stated.

### 2.2. Substrates

Three different substrates to immobilize antibodies covalently on a solid functionalized glass support were selected and compared. Two dimensional amine and epoxy modified surfaces, as well as a 3D *N*-Hydroxysuccinimide (NHS) modified hydrogel surface were selected. All slides are available from Schott (Jena, Germany) with the name Nexterion^®^ Slide A+, E, and H, respectively. All slides have furthermore a reflective optical coating to improve optical fluorescence signal to background intensities and are available under the name “HiSens”. 

**Figure 2:**
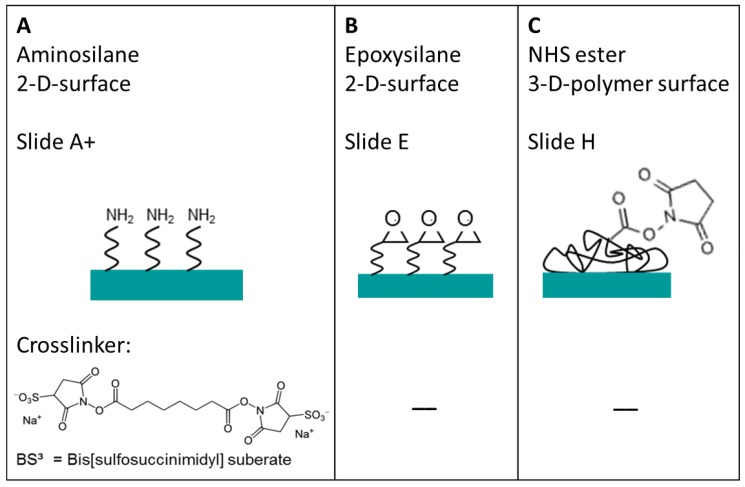
Three different functionalized glass surfaces, which are suitable for covalent protein immobilization: (**A**) amine-terminated surface; (**B**) epoxy-terminated surface; and (**C**) 3D NHS-terminated surface. In case of the amine modified surface, a crosslinker is needed to form a covalent bond. The chemical structure of the homobifunctional BS^3^ linker used in this study for the covalent immobilization of antibody to the amine modified substrate is given. In the case of the epoxy and NHS-3D surfaces a peptide bond is formed directly between surface and antibody.

The three different substrates were studied before antibody immobilization with contact angle measurements (DSA 30, Krüss GmbH, Hamburg, Germany) and X-ray Photoelectron Spectroscopy (Axis Ultra DLD, Kratos, Manchester, UK) to monitor the wetting properties as well as the quality and amount of functionalization. The functional surfaces of the glass slides are illustrated in Figure 2 together with the chemical structure of needed crosslinker.

To enable the covalent immobilization of the antibodies on the amine-modified slide, a further modification was necessary. The commercially available homobifunctional crosslinker Bis[sulfosuccinimidyl]suberate (Figure 2, BS^3^, Thermo Fisher Scientific, Rockford, IL, USA) was used at a concentration of 50 mM in phosphate buffered saline (PBS) buffer and was incubated on the slide for one hour on a shaker at room temperature. Afterwards, the slide was washed with water, dried with N_2,_ and used as the other slides (see below).

The epoxy and NHS-3D slides did not need further surface modification before spotting as the surfaces form directly a covalent peptide bond with the antibodies. The NHS-3D slide was stored at −20 °C, and was tempered to room temperature about one hour prior to use in order to avoid condensation on the substrate surface.

### 2.3. Spotting and Antibody Immobilization

For spotting the antibody-based microarray, the Nanoplotter NP2.0 non-contact spotting system from GeSiM (GeSiM, Dresden, Germany) equipped with piezoelectric nanoliter (spot volume = 400 pl) or picoliter pipettes (spot volume = 60 pl) was used. As the goal was to reduce spot size, the picoliter pipettes were used if not mentioned otherwise. The spotting pattern was defined with the software “spot front end” from GeSim. Dust-free slides were placed in the spotting system, antibody solutions and spotting buffer were placed in different wells of the microtiter plate to feed the spotting pipette. A buffer was aspirated in the pipette before the antibody solution. Fifty percent relative humidity within the spotter casing was maintained during the spotting process. 

For optimization of the spotting parameters and visualization of the spots, differently labeled TNFα and IL-6 antibodies (eBioscience) were spotted on the solid surfaces. For the cytokine detection with spotted microarray, unlabeled anti-TNFα or anti-IL-6 were spotted on the NHS-3D, HiSens. Depending on the surface and wetting properties, the center-to-center spacing varied from 100–300 µm. Up to 90,000 spots/slide (10,000 spots/cm^2^) were spotted and the incubation of protein with the surfaces for immobilization was conducted over at least one hour. After incubation the slides were visualized with the fluorescence microscope or further processed, respectively. Alternatively, the slides were stored at −20 °C. The microarray showed reactivity for at least one month. For further processing the slides were washed three times for 10 minutes with PBS buffer on a shaker and were blocked with 1M tris(hydroxymethyl)aminomethane (TRIS, Sigma-Aldrich) buffer, pH = 7 for one hour to minimize unspecific binding of the capture antibodies. The slides were again rinsed with water and dried with nitrogen.

### 2.4. Antigen Detection

When cytokines were labeled for detection, the LYNX Rapid RPE Antibody Conjugation Kit (AbD Serotec, Puchheim, Germany) was used. Therefore, the cytokine was mixed according to the manual of the supplier with a modifier reagent and incubated in the dark and at room temperature overnight with the lyophilized LYNX RPE dye. After incubation, a quencher reagent was added and the solution was ready for use on the microarray. The cytokine solution was diluted to a concentration of 3 µg/mL in 10 mM MES buffer, pH 7.8, resulting in 45 ng cytokine within 15 µL solution. Please note that RPE and PE can be considered as the same dye and are named here as PE. 

In case of detection by a sandwich assay, the PE-labeled antibody from eBioscience was pre-mixed in a vial with the cytokine and shaken in the dark and at room temperature. In detail, 100 µg/mL PE labeled anti-TNFα was premixed with 5 µg/mL TNFα in 10 mM MES buffer, pH 7.8 for at least 2 hours. In this case, 75 ng of cytokine were present in 15 µL solution.

A defined volume of 15 µL labeled cytokine or alternatively the pre-mixed cytokine with second labeled antibody was brought to the antibody microarray with a LifterSlip^TM^ 22 × 30 mm (VWR, Bruchsal, Germany), a cover slip with a spacer of ~20 µm height. The protein solution was filled between the substrate surface and the LifterSlip^TM^. The cytokine solution was left to react for at least 2 hours up to overnight in a humid, dark chamber at room temperature. Afterwards the slides were washed three times with PBS buffer, once with water, and dried with nitrogen.

### 2.5. Fluorescence Detection

To control the spotting quality and to prove antigen detection with fabricated microarrays, the slides were observed on a fluorescent microscope (Axioplan 2, Zeiss, Göttingen, Germany). The objective 20× “FLUAR” (Zeiss) was used due to its maximum light transmission and photon collection developed for demanding fluorescence applications [18]. Depending on the fluorescence label, the filter set numbers 09 (ex.: 450–490 nm, em.: 515 nm), 15 (ex.: 546/12 nm, em.: 590 nm), and 20HE (ex.: 546/12 nm, em.: 607/80 nm) from Zeiss were used. To quantify the fluorescent signals the grey value method from Zeiss software Axiovision was applied.

## 3. Results and Discussion

### 3.1. Substrate Characterization

**Figure 3:**
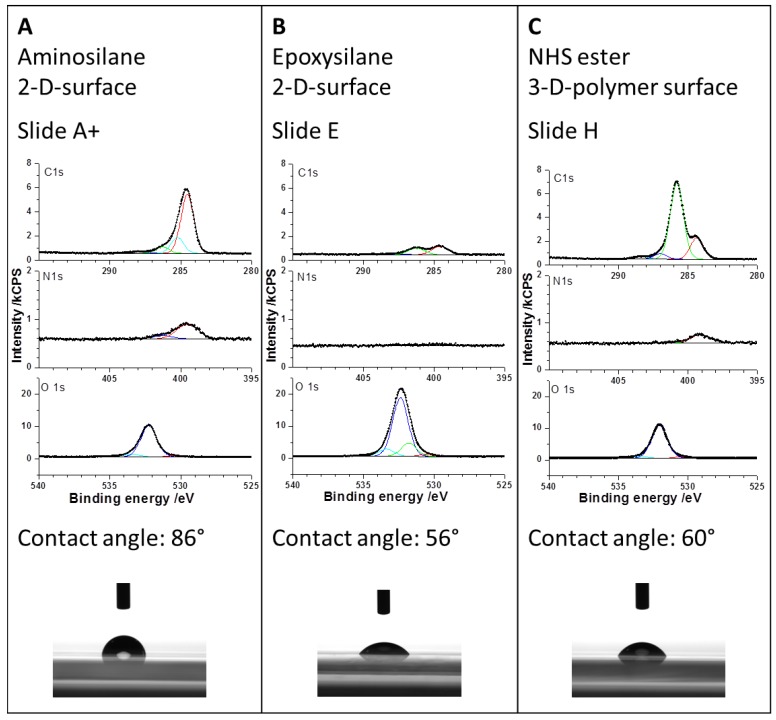
Substrate characterization of aminosilane (**A**), epoxysilane (**B**), and NHS ester (**C**) modified surfaces. Prior to antibody immobilization the substrates showed following composition as determined by X-ray photoelectron spectroscopy (XPS) and contact angles with water. N1s, C1s, and O1s XPS spectra are given for comparison.

Prior to the antibody spotting, the three different substrates amine, epoxy, and NHS-3D were characterized chemically by X-ray photoelectron spectroscopy (XPS) and physically by contact angle measurements using water in order to get a tendency for spotting behavior. The results are given in Figure 3. 

The aminosilane-functionalized slide showed the expected nitrogen due to the presence of amine groups and a high content of carbon, which was hard to judge as the structure of the aminosilane was unknown; however, suggesting the presence of alkyl chains longer than C_3_. For the immobilization of proteins it could be of advantage to choose a longer alkyl spacer unit, e.g., dodecylamine, in order to maintain the free mobility and activity of a protein. Additionally, the crosslinker’s length might also influence the antibodies’ binding activity under consideration of steric hindrances and mobility. From the XPS data it can be concluded that the alkyl chains on amine-modified slides are longer than on the epoxy-modified slides, as shown in the C1s Spectra in Figure 3. Additionally, the epoxy slide shows the expected high content of oxygen (O1s) in the substrate due to a thinner coating and a signal, which can be attributed to the epoxy group (green in Figure 3). The exact composition of the NHS-3D modified hydrogel polymer is not known, as no information from the supplier is available. The surface analysis by XPS showed a similar content in nitrogen compared to the amine slide, assuming a high modification of polymer with NHS groups. The analysis of carbon and oxygen does not give any further conclusion regarding the unknown polymer. 

Contact angle measurements with water indicate that the amine slide was more hydrophobic compared to epoxy and NHS-3D slides, resulting in a higher contact angle (86°) and hence showing the highest surface energy. Epoxy and NHS modified slides did not differ much in contact angle, showing 56° and 60°, respectively. Protein spots were expected to be smallest in diameter on the amine slides. 

### 3.2. Optimized Antibody Spot Size and Signal Density

**Figure 4:**
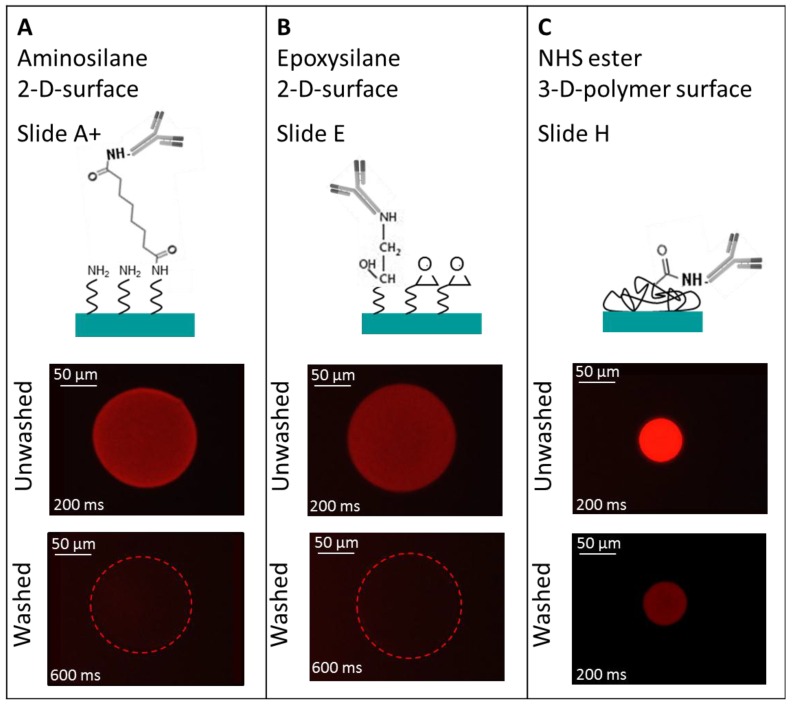
Three different selected surfaces after protein immobilization. (**A**) shows the antibody, which is immobilized via the BS^3^ linker to the amine-modified surface. (**B**) resembles the direct binding of antibodies’ amine group to the epoxy group on the surface. (**C**) the antibody binds to the NHS ester activated polymer within the 3D layer. For all surfaces there is a measured spot of unwashed and washed PE labeled anti-TNFα. Note that the fluorescence images also indicate the exposure time, showing the need to increase it from 200 ms to 600 ms for the amine and epoxy slide after washing in order to visualize the spot with the fluorescence microscope.

To obtain a high density TNFα or IL-6 antibody microarray with small, homogeneous, reproducible spots and short spot to spot distance for later sensitive cytokine detection, we visualized the spotting of fluorescently labeled antibodies on different surfaces. The three selected surfaces—amine, epoxy, and NHS-3D functionalized hydrogel substrates—were spotted with the Nanoplotter, using a picopipette and the resulting spot patterns were visualized with fluorescence microscopy. A scheme of the resulting immobilized antibodies on the surfaces together with the imaged fluorescence spots before and after washing with PBS buffer of the different slides are shown in Figure 4. Please note that in the scheme the antibody’s orientation is drawn in the oriented form for simplicity; however this does not reflect the real situation, and the antibodies are arbitrarily immobilized.

It is remarkable that the resulting spot sizes are smallest on NHS-3D slides (~50 µm) compared to the BS^3^ activated amine slide and the epoxy slide, which both have about the same diameter of ~150 µm (Figure 4). From the contact angle measurements of virgin slides, realized with water droplets (Figure 3), one would expect a slightly different result. The highest contact angle implicating a small spot size is given in case of the amino slide (86°). At the beginning, the amine terminated surface showed low hydrophilicity; it can be assumed that during the conditioning of the surface with BS^3^ in PBS buffer it becomes more hydrophilic due to the protonation of the amine groups and the interaction with the ionic BS^3^ linker. This could result in a more hydrophilic surface and hence bigger spots after antibody spotting. On the other side, the epoxy slide and the NHS-3D slide show similar contact angles of 56° and 60°, respectively, but the spot on the NHS-3D slide is much smaller with the same spotting buffer and solution. This can result from the difference in 2D and 3D surfaces, meaning that due to the 3D surface of the NHS modified slide, the layer thickness is bigger, and hence same volume of spotting solution is distributed in three dimensions spotted on a smaller area, resulting in smaller spot sizes but more intense signals compared to the 2D epoxy slide. Another explanation can be the influence of the antibody/dye solution or more likely the influence of our self-optimized buffer system depending on the functional surface. The presence of 1% glycerol possibly changes the contact angle compared to water. It is possible that the epoxy surface is more glycerol-philic compared to the NHS-3D slide with unknown polymer composition and the spotting buffer on the NHS modified slide results in a higher contact angle than with water. 

The incubation time of one hour after antibody spotting and before washing was found to be enough for all three different surfaces. This could be observed by varying the incubation time from 1–8 hours on all solid surfaces. All spots on one kind of slide time-independently showed the same intensities after washing, meaning the same grey values, regardless of whether they were incubated for one or 8 hours. It can be assumed that saturation, and hence equilibrium, is reached within 1 hour of antibody incubation time.

The fluorescent signal intensity before washing of the samples revealed the following: The PE-labeled TNFα antibody was spotted at a concentration of 25 µg/mL, meaning one single spot (V = 60 pl) contains 1.5 pg of antibody before washing. Assuming an antibody size of 150 kDa, about 6 × 10^6^ TNFα antibody molecules would be deposited within one spot before washing. On the NHS-3D slide the spots are smallest and hence show a more intense fluorescent signal compared to the epoxy and amine slide, as the 6 × 10^6^ available antibodies are more densely packed. Assuming an antibody-PE diameter d_Antibody-PE_ ~26 nm, under the consideration of d_Antibody_ ~10 nm with ~150 kDa and further d_PE_ ~16 nm for the ~240 kDa PE label, the effective surface area of a PE-labeled antibody would be ~531 nm^2^. For the densest monolayer, there is theoretically space for ~4 × 10^6^ labeled antibodies in the case of 50 µm spots as we have achieved them on the NHS-3D hydrogel surface and ~33 × 10^6^ spaces in case of 150 µm spots of the amino and epoxy functionalized supports. Admittedly, the label size plays an important role and the results will be different if no label is used for later cytokine detection. However, spotting smaller spots will result in an excess of capture antibodies for the potential immobilization of also the third dimension on the solid support, and thus a more effective binding is possible. 

On all three surfaces the spots lost intensity after the PBS washing procedure because non-immobilized labeled antibodies were washed away. Residues of antibodies from the washing process in form of non-specific binding were not observed, leading to the conclusion of an accordingly slow kinetic for the immobilization process under given parameters [19].

The ~150 µm spot on the amine surface lost its fluorescence intensity after the washing process and could only be observed with the fluorescent microscope by increasing the exposure time from 200 ms to 600 ms. A guide for the eye is given for visualization in Figure 4. It can be assumed that analyte detection in this case would lead to even weaker signals, due to a low signal to noise ratio. The amine surfaces are known to bind biomolecules, e.g., DNA, by adsorption with its negatively charged phosphate backbone to the positively charged amine surface. Alternatively, biomolecules can bind covalently to amine groups by using crosslinkers e.g., like BS^3^. The NHS groups of the BS^3^ linker, for example, leave, and the linker forms a stable peptide bond with the amine groups of the biomolecule and surface. The advantage of this relatively easy way of preparation of amine functional groups on glass or glass-like surfaces by silanization is canceled out by the disadvantage of an intermediate step with a crosslinker. At first glance, amino modified slides are not intuitive for antibody arrays, e.g., Schott recommend this surface more for DNA application, however, the contact angle measurements and promising results reported by Seurynck-Servoss *et al.* suggested an attempt. They evaluated different surfaces for antibody microarrays and found amine modified surfaces well suited regarding spot size, morphology, signal background, sensitivity and reproducibility, also in comparison with other different epoxy modified surfaces and NHS modified hydrogels [20]. The proof of antibody activity and binding capacity is not further investigated here, as we aim for small spots and a high antibody density, *i.e.*, higher signal intensities after the washing procedure.

The epoxy surface showed very similar results compared with the amine surface regarding spot size and fluorescence intensity before and after washing. For signal detection, here also the exposure time had to be increased from 200 ms to 600 ms. Epoxy groups can be attached to glass or glass-like surfaces by silanization similar to amine groups. The advantage of a time efficient direct antibody binding via an amine group of the biomolecule on the epoxy surface did not lead to better results in signal intensity.

In contrast to the amine and epoxy surface, the NHS functionalized hydrogel on a glass slide offers a 3D geometry of polymer with NHS ester groups and reacts directly with the amine group of an antibody. The main advantage of the 3D hydrogel is that it is claimed to be beneficial for protein stability, as the antibody can keep its native form and hence its activity, making it an interesting surface for antibody microarrays [14]. Additionally, the 3D setup theoretically offers more active binding sites per area, meaning a higher analyte binding capacity per spot of the same size and an increased signal. The antibody density of the ~50 µm spot after washing is still the highest in case of the NHS-3D slide compared to the epoxy slide and the amine/BS^3^ slide. This means the antibody loading is maximized for smaller spots even after washing, the final microarray can be spotted more densely, and more tests per slide can be realized. Therefore we decided to fabricate our high-density protein arrays on NHS-modified hydrogel slides. As it is still unclear how many unbound antibodies are washed away during the PBS washing procedure, the binding efficiency of immobilized antibody was estimated to be about 12%. This was done by comparing the fluorescence intensities of different antibody concentrations from 1–100 µg/mL with unwashed and washed spots (see Supplementary Materials).

**Figure 5:**
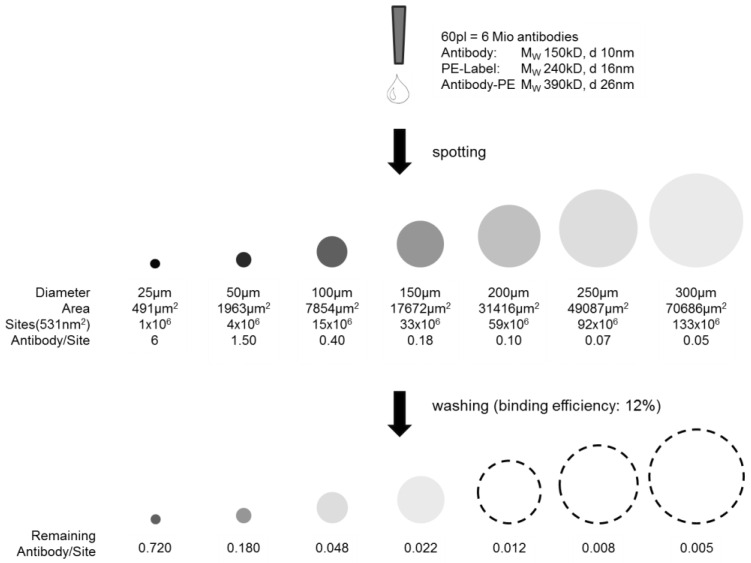
Scheme of capture antibody density per spot. Illustrated before and after washing depending on the spot size. The dashed lines are a guide for the eye, as intensities are not visible anymore.

The highest antibody density within one spot would be given if a monolayer, or in case of the 3D hydrogel slide, a multilayer with functional and active antibodies could be achieved. This may later lead to a higher signal density when detecting the analyte. A quantitative evaluation of the spotting results is given below and visualized in Figure 5. The theoretically possible antibody loading in a 50 µm spot, considering the densest monolayer, was calculated to be 4 × 10^6^ PE-labeled anti-TNFα molecules. However, the real antibody loading was estimated from the concentration line given in the Supplementary Materials: assuming an antibody concentration level of 3 µg/mL within the washed spot by comparing the fluorescent intensities of washed 25 µg/mL concentration with unwashed spots (binding efficiency ~12%), and knowing the drop volume of the nanoplotter (V = 60 pl), there are 0.18 pg antibody within one spot after washing. Assuming a molecular weight of 150 kDa per antibody and taking the Avogadro constant of 6 × 10^23^ mol^−1^, it can be concluded that the amount of antibody/spot under the given assumptions is ~7.2 × 10^5^, *i.e.*, a loading of ~18% of a theoretical dense antibody monolayer. Taking the PE dye of 240 kDa into consideration, ~47% of a theoretical dense antibody-PE monolayer is achieved. Please note that the term “monolayer” in case of a 3D surface is not appropriate but is taken for comparison and an easy imagination.

One possibility to further increase the loading of capture antibodies on the NHS modified hydrogel would be to further reduce the spot size significantly below 50 µm, which was not possible here with the given slide and instrumentation, or to increase the spotting concentration. The second option has been investigated and neglected, as high concentrations of antibodies are spotted, but also washed off afterwards due to an already good saturation of antibodies on each spot. 

Another possibility to increase the antibody loading per spot would be to exchange the fluorescent label to one of a smaller effective size, or even to utilize label-free antibodies. The protein label itself is very big (PE 240 kDa, APC 105 kDa); a small dye, for example Alexa^®^ dyes or FITC (389.2 g/mol ~0.4 kDa), could lead to higher antibody coverage and a closer approximation. The point is that the label at this stage is only used for visualization and quantification estimations before detecting the cytokine molecules. This is important to consider as density calculations show an error and most probably the pure antibody is packed even more densely on the NHS-3D slide. The theoretical optimum would be a dense layer of capture molecules; however, this is not possible due to steric hindrances and other protein-protein interactions, resulting in variable distances in-between antibodies. Additionally, it has also been reported that a highly dense antibody layer does not automatically mean that more analyte can be detected, again due to steric hindrances and interactions [14]. 

Finally, for a highly sensitive cytokine array the first step is the selection of an appropriate antibody-antigen pair with specific and high association constant *K*. The second one is, as Roger Ekins claims in his ambient analyte theory, that signal density per spot increases with smaller spot sizes till a constant level of signal density is reached, *i.e.*, the geometry and spot size reduction is of great importance for a high sensitive cytokine microarray [16,17,21,22]. Additional to Ekin’s theory, Kusnezow *et al.* [23] experimentally discussed the optimal design of microarray immunoassays, and we adapted the theory to our microarray. As illustrated and calculated in Figure 5, the estimated remaining antibody density after washing within one spot differs strongly in spot size at a constant antibody concentration spotting. The smallest spots show highest antibody density before and after washing. 

Assuming ~7.2 × 10^5^ available cytokine binding sites in a 50 µm spot (see above), an attomolar concentration, *i.e.*, 6 × 10^5^ cytokine molecules, would form a monolayer, which can potentially be detected, knowing that in practice this is difficult to achieve due to different side effects. To achieve this detection in the small spots with the calculated low analyte (antigen) concentration, the diffusion coefficient plays an important role to transport the analyte from the sample solution to the spot. The diffusion coefficient of the analyte must be very high or, as this often cannot be modified, the transport length has to be decreased in order to avoid non-specific binding and to achieve the ambient analyte status and no analyte depletion. 

### 3.3. High-Density Antibody Array

**Figure 6:**
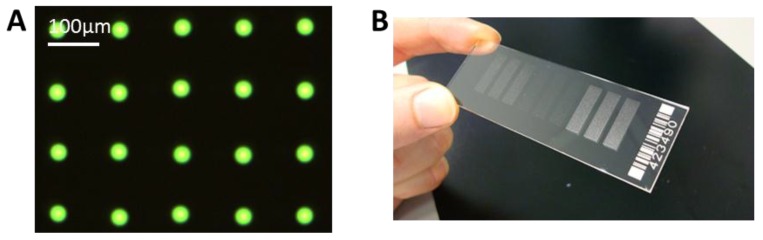
(**A**) Example of spotted microarray (before washing) with optimized parameters, 60 pl of 25 µg/mL anti-TNFα, fluorescein isothiocyanate (FITC) labeled, NHS-3D slide. The resulting spot to spot distance is 100 ± 3 µm. The spot diameter in this case is 27 ± 1 µm. (**B**) Example of ready to use antibody microarray on NHS-3D slide, nine blocks, 7500 spots each).

We have achieved a very reproducible spot size with a standard deviation of ± <1 µm for spot sizes around 30 µm. This can be seen in Figure 6A, where only one type of antibody, FITC labeled anti-TNFα is spotted. With this small and reproducible spot size, a spot to spot distance of 100 µm can be achieved, and it is possible to spot more than 90,000 spots onto a microscopic slide, with 100 spots/mm^2^.

**Figure 7:**
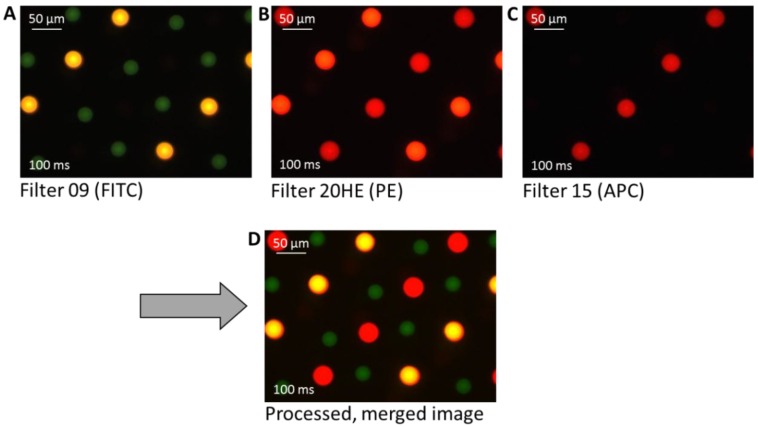
Three different fluorescence microscopy images of a fabricated multiplex cytokine array. The antibodies are differently labeled with fluorescence dyes for visualization: PE-labeled anti-TNFα (yellow-orange), allophycocyanin (APC)-labeled anti-TNFα (red), and FITC-labeled anti-IL-6 (green). The final image (**D**) shows the processed image after merging the pictures taken with different filters (**A**)–(**C**).

To explore the possibility of preparing high density multiplex arrays with high reproducibility, as well as to verify the label suitability, three different label/antibody complexes—PE-labeled anti-TNFα, APC-labeled anti-TNFα, and FITC-labeled anti-IL-6—were spotted. It can be seen in Figure 7 that also in the high density multiplex arrays the spots show good reproducibility and homogeneity in morphology and size within one type of spotted antibody. 

The unwashed spots appear to be well visible after spotting with an exposure time of 100 ms. In A, using a filter with an excitation of 450–490 nm and an emission of 515 nm (Filter 09), the FITC labeled anti-IL-6 is visible as green spots and the PE-labeled anti-TNFα appears yellow. In B, using a filter with an excitation of 546/12 nm and an emission of 607/80 nm (Filter 20HE), the before yellow PE-labeled anti-TNFα here appears orange-red, and the APC-labeled anti-TNFα is red. The FITC labeled anti-IL-6 is not visible with this filter. In C, using a filter with an excitation of 546/12 nm and an emission of 590 nm (Filter 15) only the APC-labeled anti-TNFα appears as red spots. The three taken images were merged by image processing using the computer software to depict and to differentiate between the three differently labeled cytokines within one image, the initial parameters were not modified. 

It is noted that the spot diameter of the green FITC-labeled anti-IL-6 is slightly smaller (28 ± 2 µm) than that of the other labeled TNFα spots (PE 37 ± 2 µm, APC 41 ± 2 µm). One reason for the slight deviation of the spot size could be that the cytokines themselves have different physico-chemical properties and structures, which will result in different ways of interacting with the surface. A further reason could be that FITC is a rather hydrophobic label. Additionally, it could be an optical effect, as FITC bleaches faster than PE or APC and hence the FITC spot appears slightly smaller. In summary, the same spotting pipette, the same substrate, and the same dilution buffer result in very reproducible spots of the same size, only the changing antibody/dye/additives show an influence on spot size, and we could observe differences of about 10–20 µm in diameter. Furthermore, by spotting such a mixed array, it is noted that the position of the spots is more difficult to control compared to single antibody solution, which is limited by the precision of the instrument.

### 3.4. Analyte Detection

Direct assay: The antibodies are receptor molecules selected to target cytokines (here: TNFα and IL-6) as disease biomarkers. Those cytokines occur in exhaled breath condensate and can potentially give a statement of COPD and asthma levels in patients. The analyte detection clarifies if the antibody microarray can potentially fulfill the detection of low concentrated biomarkers. 

**Figure 8:**
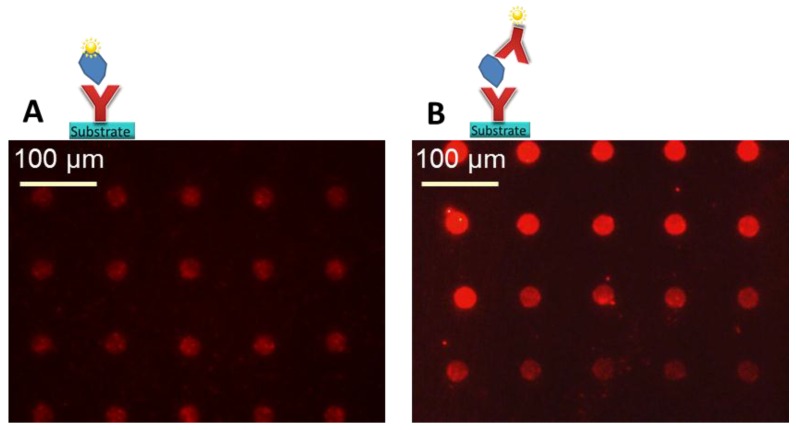
Original fluorescence images. (**A**) Direct assay, detection of ~0.6 pg IL-6 per spot on a prepared anti-IL-6 microarray, assuming 100% label efficiency with PE. (**B**) Sandwich assay, 5 µg/mL TNFα was pre-incubated with 100 µg/mL PE-labeled anti-TNFα. Detection of ~1 pg TNFα per spot on a prepared anti-TNFα microarray. A concentration gradient from border to center becomes visible in this experiment. Note: only a representative part of the whole array is depicted.

To prove the analyte detection with the prepared high-density arrays, the antigen was directly labeled with a fluorescence dye. The labeling efficiency of the used labeling kit was not analyzed, a 1:1 ratio of cytokine to PE label was assumed according to supplier’s information. This means, it has to be considered that if the labeling efficiency is not 100%, not all bound cytokines can be optically detected by fluorescence microscopy, resulting in an effectively even higher sensitivity. The previously labeled cytokine solution (3 µg/mL) reacted in the microarray with a defined volume, given by the LifterSlip^TM^ geometry. After reaction of the labeled antigen with the unlabeled, spotted antibody array and washing to remove unbound label as well as unbound antigen, we observed the fluorescent signal shown in Figure 8A. We chose a low µg/mL concentration for the first experiments in order to be able to expect a visible fluorescence result with the microscope. This means here 45 ng of cytokine was available for binding to the 66,000 spots in the array below the 22 × 30 mm LifterSlip^TM^. When the geometry of the array is taken into account, as in a conventional antibody array, we will only have e.g., single spots or more likely triplicates of one type of antibody, hence we determined that one single spot has a detection volume of 0.2 nl/spot (100 µm × 100 µm × 20 µm—spot to spot distances and height of the LifterSlip^TM^). Thus, if 3 µg/mL IL-6 (M_W_ = 20 kDa) can be detected with the antibody microarray, one single spot detected ~0.6 pg IL-6 (=30 attomoles or ~1.8 × 10^7^ molecules). Considering that if just the spot to spot distance is doubled within the same setup, the detectable mass of a single spot at the same concentration is at ~2.4 pg. As the spot offers ~7.2 × 10^5^ binding sites, only 4% of the cytokine molecules are able to find corresponding binding partners. This calculation reveals that with this method, *i.e.*, by direct analyte labeling, it is possible to detect pure analyte in the low pg range/spot, having a µg/mL sample concentration, and that the cytokine concentration can be potentially further reduced. The incubation overnight showed to be beneficial for signal intensities. Long incubation times were also suggested by Krusnezow *et al.* [23]. 

Sandwich assay: Alternatively, the analyte was detected by a second labeled antibody in the sandwich assay format, even the PE labeled anti-TNFα was premixed with TNFα, which made it possible to minimize the incubation on the microarray to one step. We saw that this procedure showed less non-specific binding and a higher signal to background as if the two steps were realized on the antibody microarray one after the other (data not shown). The ratio of PE: anti-TNFα was 1:1 according to the supplier’s information. The premixed solution with a cytokine concentration of 5 µg/mL reacted with a defined volume on the microarray. This means that 75 ng of cytokine were available for binding to all spots. After reaction with the unlabeled, spotted antibody array and washing, fluorescent signals as shown in Figure 8B resulted. Taking again the geometry of the array and the 66,000 spots into account, (V = 0.2 nl/spot), it can be calculated, that if 5 µg/mL TNFα (M_W_ = 17 kDa) can be detected with the antibody microarray, one single spot detected ~1 pg TNFα (=60 amol or ~3.6 × 10^7^ molecules). As the spot offers ~7.2 × 10^5^ binding sites, only 2% of the cytokine molecules are able to find corresponding binding partners. The calculation reveals again that with this method it is also possible to detect the cytokine in the low pg range/spot in a µg/mL sample concentration, and that the cytokine concentration can be potentially further reduced.

The blocking of the NHS modified surface by an amine-containing buffer, 1M TRIS, resulted as suitable in all experiments; however, non-specific binding was still present. Alternatively, the blocking buffer recommended by the supplier of the hydrogel slide worked well for given slide, but should be avoided because of carcinogenic, mutagenic and toxic to reproduction (CMR) containing material. Lower TRIS buffer concentrations, like 20 mM or 150 mM showed an increased non-specific binding (data not shown).

It was shown that the antibody microarray works and both ways of detection (direct and sandwich assay) result in similar findings. As we wanted to further illustrate the proof of principle in detection with the fluorescent microscope, we chose to run the experiments at a higher cytokine concentration in the low µg/mL-range; detection was even possible in the ng/mL range (data not shown, weaker fluorescence signals). The determination of the LoD at this stage was not yet targeted. However, during all experiments, it was observed that the signal intensity was not equal over the whole microarray, which is clearly visible in Figure 8B. The spots at the border of the microarray and LifterSlip^TM^, where the solution was inserted manually with the pipette, showed stronger signals than those in the center of the array. This also applied if the incubation was over night, allowing more time for the reaction. One possibility could be the depletion of analyte concentration when applying the sample from the edges of a slide with a pipette. This would mean that the spot in the upper row (left) detects the initial cytokine concentration and towards the bottom (right) we approach the LoD, without knowing this final concentration. In the first instance, the reason for analyte depletion is the amount of antibody because the high spot density array of the same antibody offers many specific binding sites, similar to a larger sized spot, leading to depletion of the analyte in the solution. The observation leads, as expected, to the conclusion that ambient analyte conditions, as described by Ekins do not yet apply for this microarray, as the assay is not independent of sample volume and nor of the present amount of antibodies. However, in a real assay, one would of course not target an array with such a spot density, as more than a triplicate per type of antibody is usually not needed. This layout was only selected to demonstrate the spot density. The second point is that Ekins does not consider the unspecific binding, which always causes analyte depletion, especially for low analyte concentrations. This means, to approximate Ekins theory, higher optimization will be needed, considering for example the measurement of the diffusion constants. Due to the analyte depletion from the edges towards the center of the array, the feeding of the target analyte onto the point of detection is of importance. A promising solution could be the spotting of the analyte spot directly onto the detection spot. Assuming that the sample is not in contact with any material, which unspecifically binds the proteins, this would lead to a minimal loss of sample volume, a minimum of unspecific binding and a minimum in diffusion length for protein-protein binding. Spotting of sample on the antibody microarray using a nanoplotter is unfortunately not user friendly, this is why a design of microfluidics under consideration of gained knowledge becomes interesting. Work in this direction to combine an antibody microarray with microfluidics is ongoing and similar work is reported by different groups [13,24,25,26].

## 4. Summary and Outlook 

In summary, a high-density antibody microarray for a potential cytokine detection was fabricated, and several considerations for a further development towards a sensitive antibody microarray were concluded. Suggestions were made in order to detect at a later stage target analyte in body fluids, for example in exhaled breath condensate. As postulated by Ekins, small spots showed a positive effect on the analyte detection. In the present study, small, compact, homogeneous, and reproducible protein spots were realized by using a non-contact printing nanoplotter with picopipettes, as these spot characteristics were unfortunately not automatically given when looking at antibody microarrays which were available on the market. Additionally, the optimization of the antibody spotting buffer to avoid salt formation and inhomogeneity was realized and contributes to the above mentioned spot characteristics. The spot size and spot to spot distance were reduced according to the potential of the selected solid support and the spotting equipment. Tendencies of the wetting properties of the antibody spotting solution on the solid support can be given by contact angle measurements; however this does not replace the real spotting experiment for final conclusions. In our case, the best results were achieved with the commercially available NHS-3D slide, HiSens, which allows a direct and covalent antibody immobilization without further reaction steps. By applying optimized spotting parameters, the spot size was reduced down to ~30 µm with ~100 µm spot to spot distance. This geometry differs from many other antibody microarrays with spot sizes of >100 µm and with a spot to spot distance of >250 µm. In the literature, lithographic technologies to fabricate arrays with spot sizes down to 50 nm are undergoing development in order to reduce sample volume, incubation time, and possibly the LoD [15]. However, in this case it would be necessary to change experimental parameters and conventional fluorescent scanners or microscopes, which would be another topic of investigation. Our low spot to spot distance is of advantage, considering the diffusion and kinetics of proteins in a microarray immunoassay. Short diffusion lengths were further realized by using the LifterSlip^TM^. Analogous considerations like microfluidics or similar approaches should be taken into account for further developments. For the alternative amine and epoxy functionalized surfaces, it was also possible to bind antibodies covalently; however further investigations were omitted because of their drawbacks. On these surfaces the spots were ~3× bigger, the antibody could not be as densely packed, and fluorescent intensities of spotted antibodies were already very low after antibody washing, and minimal higher antibody density could only be achieved if the concentration of antibody was increased significantly. Additionally, the stability of the fabricated antibody microarray on the 3D hydrogel could be given at least for 1 month, meaning no significant quality loss could be observed.

Further, we investigated different fluorescence dyes, of which APC and especially PE showed very high fluorescent intensities; however, as they are themselves large proteins they might influence the wetting properties, the coverage, as well as the antibody-antigen binding process, *i.e.*, the biological activity. The FITC label was smaller but on the other hand not suitable due to strong bleaching. Stable and small labels, ideally with inert properties could reduce the effects of a label to a minimum, reducing steric hindrances and other systematic errors within the protein-protein binding. 

To prove the function of the fabricated antibody microarray, the analyte was detected by either direct cytokine labeling or by a second labeled antibody. In both cases the amount of cytokine detected by a single antibody spot was in the low pg-range, and this without further optimization of the LoD. Apart from the detectable mass, the sample volume was also of importance and the interesting question was: what is the absolute mass detected by a single spot within a microarray? Achieved attomolar sensitivity of one single spot is comparable to the golden standard method for cytokine detection, the ELISA, however, the microarray has the potential to be geometrically optimized towards even higher sensitivities, independently from the fact that high sensitivity is given by the perfect antibody-antigen partner. To further improve the LoD in the future, different signal amplification techniques as for example reactions catalyzed by horseradish peroxidase (HRP) as known from the standard ELISA, or PCR-based amplification techniques can be considered in combination with an appropriate blocking procedure to further reduce background signal and unspecific binding. 

The vision is still to have a pattern of relevant and validated biomarker molecules, which can possibly give more information regarding a disease or therapy monitoring. In clinical diagnostics there are still many challenges to overcome in order to develop tools for clinically relevant and validated biomarkers. It is unknown how many discovered biomarkers fail in the validation process, and a single biomarker may lack in specificity and fail FDA approval [15]. If thinking about a diagnostic device, 100 spots/mm^2^ can already result in thousands of assays on an area of a microscopic slide. The vision to be very sensitive, to avoid sample pre-treatment, and to detect sample volume and concentration independently might become possible within a small volume microfluidic device with negligible non-specific binding, also for whole sample of body fluids. A mixture of miniaturized ELISA and antibody microarray for low LoDs could be the next generation immunoassays, reducing sample volume, unspecific binding, and different kinds of cross-reactions in multiplexing [13].

## Supplementary Files

Supplementary File 1
