# Supplementary material for: Fabrication of Homogeneous High-Density Antibody Microarrays for Cytokine Detection
Source: Microarrays (Basel). 2014 Dec 9;3(4):282–301. doi: 10.3390/microarrays3040282 (PMC4979058; doi:10.3390/microarrays3040282)

Supplementary Materials

Fabrication of Homogeneous High-Density Antibody Microarrays for Cytokine Detection

Ingeborg Hospach, Yvonne Joseph, Michaela Kathrin Mai, Nadejda Krasteva and Gabriele Nelles

S1. Selection and Optimization of Buffer System

For the optimization of the spot size and morphology regarding homogeneity and reproducibility, 200 ng/mL fluorescein were dissolved in 10 mM phosphate buffer (CAS 10049-21-5) and spotted with varied glycerol (CAS 56-81-5) concentrations in the range of 0–5% and varied sodium chloride (NaCl, CAS 7647-14-5) salt concentrations in the range of 0–125 mM to find the appropriate concentrations. Glycerol is added to spotting buffers in order to prevent the total dry out of the protein on the microarray and therefore to increase the shelf life of the antibody. The spotting of this buffer was tested with the Nanoplotter, equipped with a nanoliter pipette on an ethanol cleaned microscopic glass slide form VWR (Bruchsal, Germany). Exemplary spots are shown in Figure S1.

Figure S1. (**A**) 10 mM sodium phosphate buffer with varied concentrations of glycerol. 1% glycerol produced best spot morphology and avoids total dry out of the proteins. (**B**) 10 mM sodium phosphate buffer with 1% glycerol and varied NaCl concentrations. Presence of NaCl results in salt crystals that cause inhomogeneous spot brightness. No NaCl resulted in best spot morphology with almost ideal homogeneous brightness throughout the spot area.

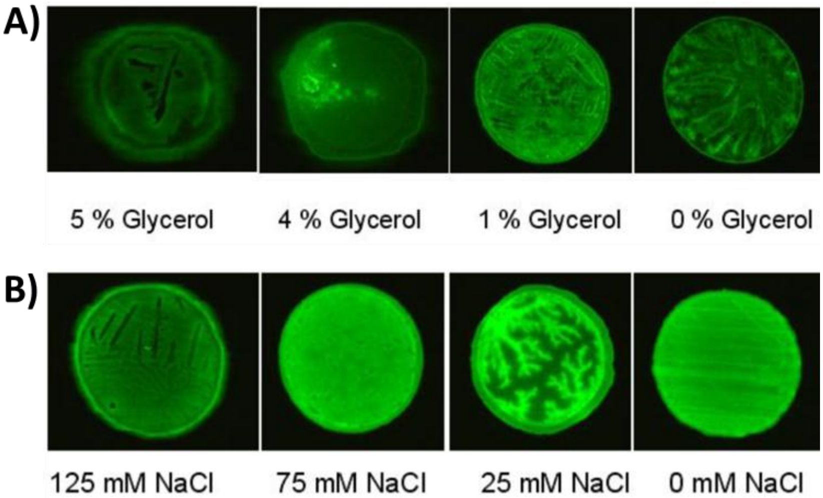

1% glycerol was defined as appropriate as the spots are more homogeneous as in the case of 4% and 5% glycerol concentration. High concentrations of glycerol results additionally in diffuse edges and the absence of glycerol is not advantageous regarding proteins stability. Low salt concentrations resulted to be beneficial as crystal formation is avoided. We observed that crystal formation has an influence of the homogeneous and reproducible antibody interaction with the solid substrate. Therefore no additional NaCl is added to the spotting buffer.

Our optimized buffer system was additionally compared with the commercial spotting buffer Nexterion® Spot PB (Schott, Jena, Germany) on the solid support Nexterion® Slide H (Schott). With both buffers a TNFα antibody concentration of 10 µg/mL was prepared (APC labeled, eBioscience, Frankfurt am Main, Germany) and spotted the same way with the Nanoplotter (GeSiM, Dresden, Germany) using a picopipette. As it can be seen in Figure S2 the spot has a smaller size (~60 µm) when using our buffer system compared to the spot when using the commercial buffer (~190 µm spot size). As we want to achieve small spots we defined our buffer system as more suited for our purpose and used it for all further experiments if not other mentioned.

Figure S2. Comparison of commercial spotting buffer from Schott with our spotting buffer regarding spot size. 10 µg/mL anti-TNFα–APC was spotted. Spots resulted to be smaller when the 10 mM phosphate buffer, 1% glycerol is used. The fluorescence was observed with the fluorescent microscope from Zeiss. The intensities and hence the antibodies concentration decreased after washing as expected and therefore a guide for the eye is given for visualizing the washed spots.

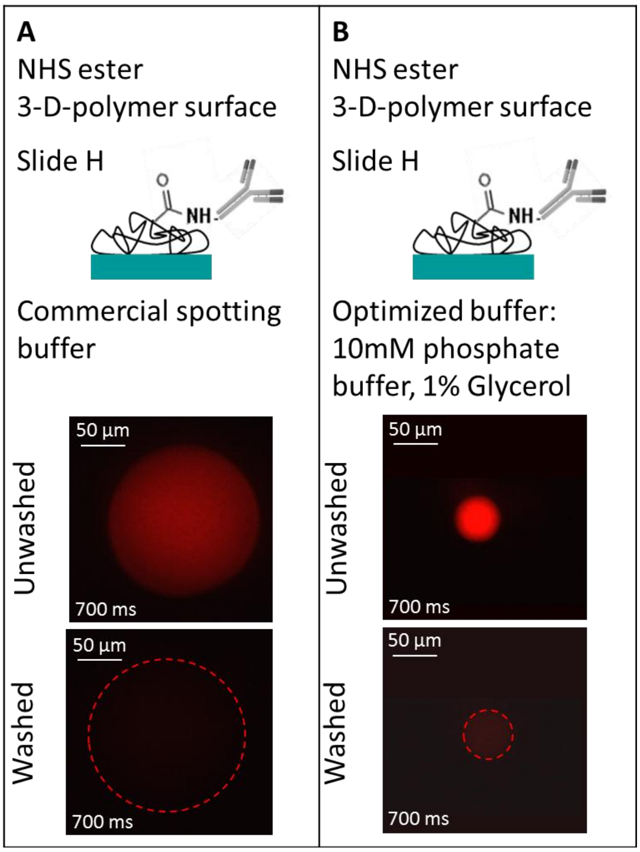

S2. Concentration Line

To estimate the appropriate antibody concentration for spotting of a microarray and to estimate the amount of bound antibodies per spot (spot density), a concentration line from 0–100 µg/mL labeled antibodies in optimized spotting buffer were spotted on Slide H, HiSens and fluorescent images were taken. After the washing steps with PBS, images were again taken and the fluorescent intensities (grey values) of spotted concentration range was compared by using the Zeiss software. The grey value can

be used for intensity measurements, as the grey value is the brightness value of a pixel. Here the average grey value of a spot was determined by selecting the spot area. The defined area can be used as a reference for following spots and for comparison.

In Figure S3 it can be observed in the lower row that after washing antibody saturation on the slide seems to be achieved for higher concentrations, assuming that it is not possible to bind much more labeled capture analyte on the hydrogel surface, even if a higher concentration is offered. An appropriate antibody concentration for spotting was defined. The grey values of the different spots of the concentration line are also illustrated in Figure S4, where the graph becomes more flat for higher concentrations, even a real saturation could not be achieved below 100 µg/mL antibody. Under the consideration of resources and the price of antibodies, we defined a 25 µg/mL antibody concentration for spotting as a good compromise, because good results, *i.e.*, nicely visible fluorescent signals could be achieved. After washing the spots of 25 µg/mL, the intensity is similar to estimated 3 µg/mL of unwashed protein, resulting in a binding efficiency of ~12%.

Figure S3. PE labeled anti-TNFα at a concentration of 1–100 µg/mL in spotting buffer was spotted on the 3D Slide H, HiSens from Schott. In the upper row the unwashed spots are depicted, in the lower row the spots after the PBS washing procedure are depicted. Note that all spots are exemplarily and it is not the same spot before and after washing, just from the same slide. The arrow indicates the concentration range after washing, *i.e.*, ~3 µg/mL is left after washing the 25 µg/mL spot.

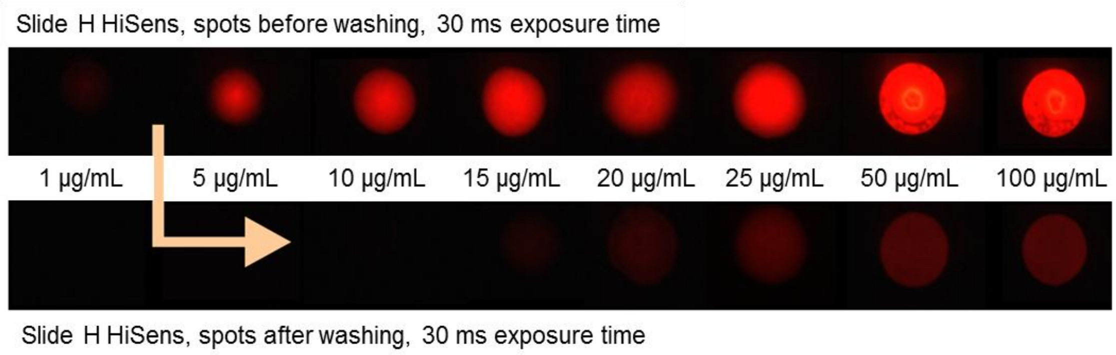

Figure S4. By the grey value detection with the Zeiss software, the range of saturation after washing could be assumed. Even no total saturation could be seen below 100 µg/mL, we defined 25 µg/mL as a well suited concentration for spotting, considering spot density and ecology.

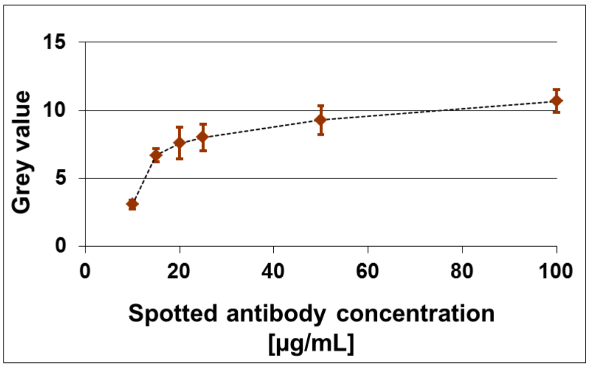

Supplement: Supplementary File 1 [file microarrays-03-00282-s001.pdf]
